# Treating chronic obstructive pulmonary disease with ensifentrine: a systematic review

**DOI:** 10.3389/fmed.2025.1595662

**Published:** 2025-09-05

**Authors:** Sultan Almuntashiri, Moaddey Alfarhan, Aaron Chase, Xiaoyun Wang, Duo Zhang, Arshad Hussain, Heba Ali Khloofi, Ali Alghubayshi, Sirajudheen Anwar

**Affiliations:** ^1^Department of Clinical Pharmacy, College of Pharmacy, University of Hail, Hail, Saudi Arabia; ^2^Department of Clinical Practice, College of Pharmacy, Jazan University, Jazan, Saudi Arabia; ^3^Department of Clinical and Administrative Pharmacy, College of Pharmacy, University of Georgia, Augusta, GA, United States; ^4^Clinical and Experimental Therapeutics, College of Pharmacy, University of Georgia and Charlie Norwood VA Medical Center, Augusta, GA, United States; ^5^Department of Pharmacology and Toxicology, College of Pharmacy, University of Hail, Hail, Saudi Arabia

**Keywords:** chronic obstructive pulmonary disease, COPD, ensifentrine, phosphodiesterases, PDEs

## Abstract

**Background:**

The cornerstone medications for maintenance of chronic obstructive pulmonary disease (COPD) have remained the same for decades. Despite combination therapy with multiple mechanisms of action, patients with COPD have significant morbidity and frequent exacerbations. New treatments with novel mechanisms of action are needed to decrease exacerbation and improve symptoms. Ensifentrine is a novel dual PDE 3 and 4 inhibitor emerged and established as a promising drug in the treatment and management of COPD.

**Objectives:**

The purpose of this study was to examine the pooled efficacy and safety of ensifentrine versus placebo for treatment of moderate to severe COPD.

**Data sources:**

We explored PubMed, MEDLINE, and Cochrane Library databases.

**Study eligibility criteria:**

Randomized controlled clinical trials (RCTs)comparing ensifentrine 3 mg twice daily to placebo for treating moderate-to-severe COPD were included.

**Design and method:**

A systematic review of three RCTs investigating the use of ensifentrine in adults with moderate to severe COPD was performed. Mean and risk differences with 95% confidence intervals (CI) were used to express the pooled effect on continuous and binary outcomes, respectively.

**Results:**

This systematic review included data from three randomized controlled trials encompassing a total of 1,715 patients. Of these, 1,057 patients received ensifentrine and 658 received placebo. Ensifentrine was associated with significant improvements in all primary outcomes compared to placebo. The pooled mean differences in peak FEV₁, average FEV₁, and morning trough FEV₁ were 143.91 mL, 91.71 mL, and 43.69 mL, respectively (all *p* < 0.05). Regarding secondary outcomes, ensifentrine significantly improved respiratory symptom scores assessed by the Evaluating Respiratory Symptoms in COPD (E-RS: COPD) tool (*p* = 0.02), as well as the Transition Dyspnea Index (TDI) score (*p* < 0.001). The incidence of adverse events was comparable between the ensifentrine and placebo groups.

**Conclusion:**

Ensifentrine consistently improved pulmonary function tests and symptom scores with a safe adverse effect profile. This systematic review supports the clinical benefits of ensifentrine in patients with moderate to severe COPD.

## 1 Introduction

Chronic obstructive pulmonary disease (COPD) is characterized by progressive and irreversible airflow obstruction, airway remodeling, persistent inflammation, and excessive mucus secretion which lead to daily symptoms that affect quality of life (1). The disease is driven by complex pathological mechanisms involving oxidative stress, protease-antiprotease imbalance, and prolonged immune cell activation that damages lung parenchyma and narrows airways (2–4). Chronic inflammation not only drives mucus hypersecretion and smooth muscle dysfunction but also contributes to progressive airway remodeling, a feature shared with other obstructive airway diseases such as asthma (5).

Current treatments for the maintenance of COPD include long-acting beta agonists (LABAs), long-acting muscarinic antagonists (LAMAs), inhaled corticosteroids and other short-acting therapies for symptom control (6). Many patients with moderate to severe COPD remain symptomatic with frequent exacerbations and reduced quality of life despite maximal doses of combination therapy (6, 7). Treatments with novel mechanisms of action that provide better disease control without serious side effects are urgently needed to decrease the morbidity associated with the disease. Dual phosphodiesterase inhibitors have recently emerged as a potential therapeutic option (8).

Phosphodiesterases (PDEs) are enzymes that regulate a range of cellular functions including smooth muscle relaxation and inflammation by modulating cellular concentrations of cyclic nucleotides (9). PDE3, for instance, regulates both cyclic adenosine monophosphate (cAMP) and cyclic guanosine monophosphate (cGMP) levels in airway smooth muscle, whereas PDE4 regulates cAMP in cells associated with airway inflammation. Inhibition of PDE3 and PDE4 results in airway smooth muscle relaxation and anti-inflammatory effects, respectively (10). Increasing evidence suggests that combined inhibition of PDE3 and PDE4 provides synergistic effects, making this paired mechanism of action a promising strategy for COPD treatment (11, 12).

Ensifentrine is a selective dual inhibitor of PDE3 and PDE4. Previous clinical evidence has demonstrated both bronchodilatory and anti-inflammatory effects in healthy volunteers and individuals with COPD when treated with nebulized ensifentrine (13–15). Studies have also shown improvements in symptoms, lung volumes, and lung function tests when ensifentrine was used in combination with other bronchodilators (14, 16). In June 2024, ensifentrine inhalation was approved for the maintenance treatment of COPD in adults in the United States (17). Here, we aimed to examine the overall efficacy and safety of ensifentrine versus placebo using three independent clinical trials.

Several systematic review and meta-analyses have recently examined the efficacy and safety of ensifentrine in patients with COPD, reflecting growing interest in its potential role as a dual bronchodilator and anti-inflammatory agent (18–20). These studies have provided valuable insights into its clinical benefits; however, variations in outcome definitions, dosing analyses, and data presentation have limited the clarity and comparability of findings. Consequently, there remains a need for a more structured synthesis of the available evidence, particularly one that clearly categorizes outcomes and emphasizes clinically relevant dosing. This study seeks to address these gaps and provide a comprehensive, clinically meaningful evaluation of ensifentrine’s therapeutic effects in COPD.

## 2 Methods

In order to perform this systematic review, the Preferred Reporting Items for Systematic Reviews and Meta-Analyses (PRISMA) standards were followed. These guidelines ensure transparency and methodological rigor in the identification, selection, and reporting of included studies (21).

### 2.1 Search strategy

**Figure 1:**
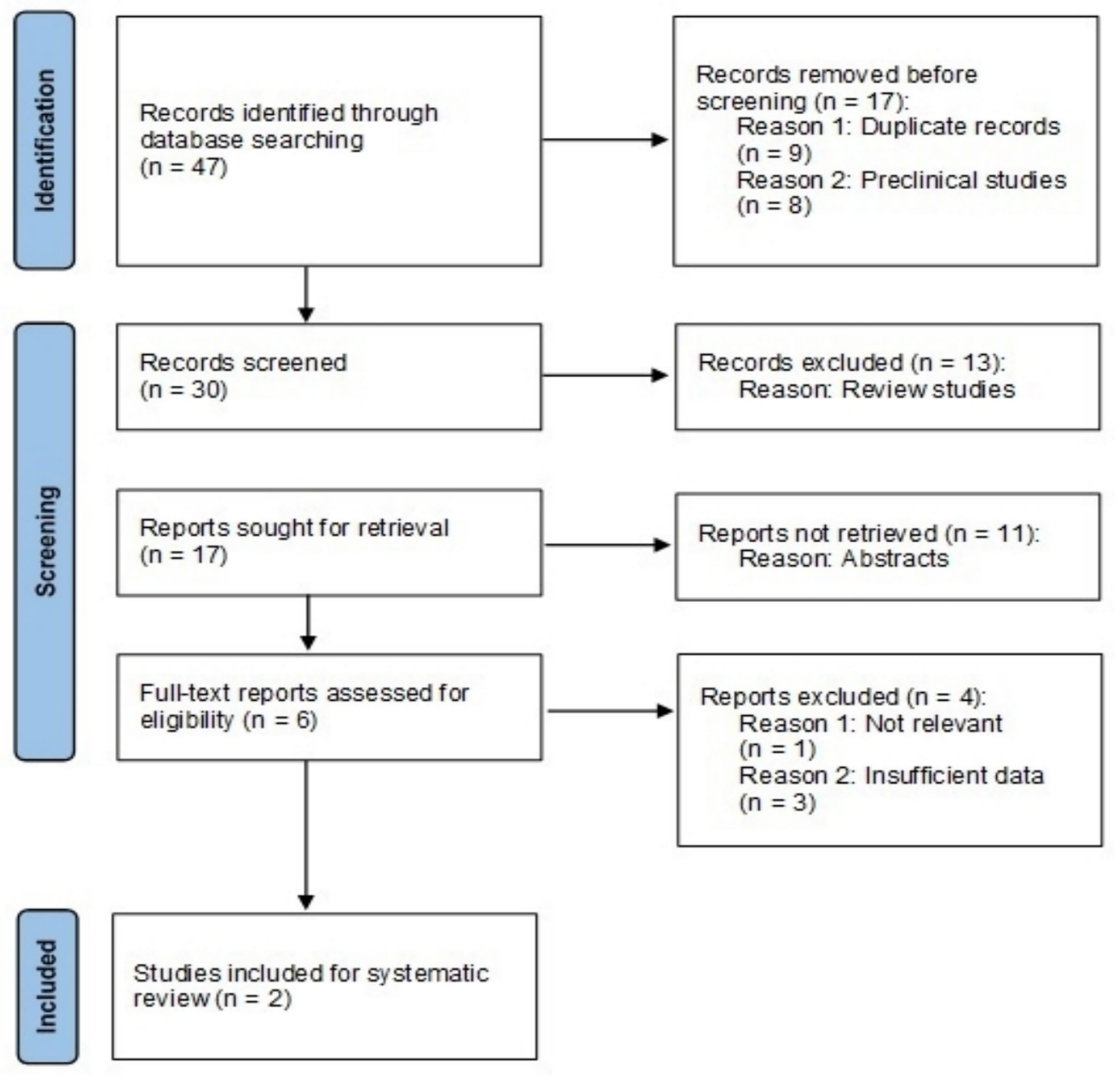
Search strategy and study selection.

A comprehensive search in PubMed, MEDLINE, and Cochrane Library databases was performed using the following keywords: “chronic obstructive pulmonary disease,” “COPD,” and “Ensifentrine” from inception to October 2024. The publication type of randomized controlled clinical trials (RCTs) was strictly imposed. We reviewed the literature and manually searched any related article to determine all eligible studies and minimize potential bias. The search strategy is described in Figure 1.

### 2.2 Inclusion and exclusion criteria

The inclusion criteria included (1) RCTs; (2) moderate-to-severe COPD adult population; (3) intervention treatments limited to ensifentrine 3 mg twice daily in comparison to placebo; (4) studies with complete full-text; and (5) studies reported in English language. The exclusion criteria included (1) studies comparing ensifentrine to placebo in asthma; (2) cohort or case control studies; (3) preclinical studies; (4) review studies, conference papers or editorial articles; (5) duplicate studies; and (6) studies with insufficient or irrelevant data.

### 2.3 Study selection

Two authors independently reviewed the search results and evaluated the eligibility of the studies for selection. Any disagreement was resolved by discussion and a third author was arbiter. After proper searching and discussion, we included three randomized, double-blind, placebo-controlled trials in this systematic review.

### 2.4 Primary, secondary, and safety outcomes

The primary outcome was the pooled efficacy of ensifentrine compared to placebo for pulmonary function tests including peak forced expiratory volume in one second (FEV_1_), average FEV_1_, and morning trough FEV_1_. Secondary efficacy outcomes included pooled analysis of patient reported respiratory assessment scores. Safety outcomes were the incidence of treatment-emergent adverse events (TEAEs), TEAEs leading to drug discontinuation or death, COPD exacerbation, hypertension, and nasopharyngitis.

### 2.5 Data extraction

Two independent investigators extracted the desirable information from each enrolled study including information related to study design, population, intervention treatments, follow-up period, outcome measures, and study results. Different opinions between the two investigators were resolved by discussion or consulting a third investigator. All desirable data were pulled from available published articles.

### 2.6 Quality assessment

The Cochrane risk of bias tool was used in evaluating the methodological quality (22). Each study was assessed for selection bias (random sequence generation and allocation concealment), reporting bias (selective reporting), blinding bias (participants, personnel, and outcome assessment), attrition bias, and other bias. Two authors reviewed all studies and assigned a value of ‘high’, ‘low’, or ‘unclear’ to each bias assessment.

### 2.7 Data analyses

Mean difference with 95% confidence interval (CI) was used to express the pooled effect on continuous variables. Risk difference with 95% CI was used to express the pooled effect on binary outcomes. Heterogeneity across included studies was assessed using the *I*^2^ statistic. Differences were considered statistically significant at *p* < 0.05. All analyses were performed with STATA version 18.0 (Stata Corp, College Station, TX, United States).

## 3 Results

### 3.1 Search strategy

Forty-seven reports were found through the initial search. Seventeen were excluded due to duplication or because they involved preclinical research. Thirty papers were screened, and 24 were excluded as they were reviews or abstracts only. Six potentially relevant trials were identified for full-text reading; however, four trials were excluded due to insufficient data or lack of relevance to our analysis. Finally, two papers were selected for the systematic review which included three RCTs (Figure 1).

### 3.2 Patient characteristics

This systematic review included data from three randomized controlled trials involving a total of 1,715 patients, with 1,075 receiving ensifentrine and 658 receiving placebo. The average age was 64.7 years old (SD, 7.6 y) with 815 (49%) females. Baseline characteristics and parameters including study design, inclusion/exclusion criteria, clinical outcomes, and follow-up period are shown in Table 1.

**Table 1:** Baseline characteristics and parameters of the included studies.

Parameter		Ferguson et al. (23)	Anzueto et al. (24)ENHANCE-1	Anzueto et al. (24) ENHANCE-2
Study design		RCTs	RCTs	RCTs
Inclusion criteria		40–80 years old COPD diagnosis Post-bronchodilator FEV_1_ 30–70% Predicted normal FEV_1_/FVC < 0.7 ⩾2 mMRC dyspnea scale score Smoking history ⩾10 pack-years		
Exclusion criteria		Patients with asthma		
Primary outcomes		Peak FEV_1_ Average FEV_1_ (0–12 h) Morning trough FEV_1_		
Secondary outcomes		SGRQ total score E-RS: COPD total score TDI score		
Follow-up period		4 weeks	12 and 24 weeks	12 and 24 weeks
No. of patients	E	82	477	498
	P	84	283	291
Female, *n* (%)	E	45 (54.9)	203 (42.6)	254 (51.0)
	P	44 (52.4)	116 (41.0)	153 (52.6)
Age, mean (SD)	E	64.5 (7.92)	65.1 (7.1)	65.0 (7.4)
	P	63.6 (8.41)	64.9 (7.7)	65.3 (7.3)
Post-bronchodilator FEV_1_, % predicted normal (SD)	E	50.4 (10.61)	52.9 (10.3)	50.8 (10.7)
	P	48.9 (10.93)	51.7 (10.5)	50.4 (10.7)
History of COPD exacerbation, *n* (%)	E	–	120 (25.2)	102 (20.5)
	P	–	75 (26.5)	62 (21.3)
Smoking history, *n* (%)				
smokers	E	43 (52.4)	268 (56.2)	276 (55.4)
	P	53 (63.1)	163 (57.6)	160 (55.0)
Former smokers	E	39 (47.6)	209 (43.8)	222 (44.6)
	P	31 (36.9)	120 (42.4)	131 (45.0)
Mean pack-years (SD)	E	51.0 (20.56)	41.1 (20.7)	42.7 (22.9)
	P	52.5 (27.37)	41.8 (20.6)	41.9 (20.9)
Prior or concomitant COPD medication, *n* (%)				
LABA	E	0	89 (18.7)	34 (6.8)
	P	2 (2.4)	45 (15.9)	23 (7.9)
LAMA	E	32 (39.0)	151 (31.7)	168 (33.7)
	P	43 (51.2)	76 (26.9)	90 (30.9)
LABA/ICS	E	5 (6.1)	87 (18.2)	72 (14.5)
	P	13 (15.5)	66 (23.3)	47 (16.2)
LAMA/ICS	E	–	4 (0.8)	1 (0.2)
	P	–	5 (1.8)	0

FEV_1_, forced expiratory volume in one second; FVC, forced vital capacity; ENHANCE: Ensifentrine as a Novel Inhaled Nebulized COPD Therapy; RCT, randomized controlled trials; E, ensifentrine; P, placebo; mMRC, modified Medical Research Council dyspnea scale score; SGRQ, St. George’s Respiratory Questionnaire total score; E-RS: COPD, evaluating respiratory symptoms in COPD total score; TDI, Transition Dyspnea Index score; LABA, long-acting beta agonist; LAMA, long-acting muscarinic antagonist; ICS, inhaled corticosteroid.

### 3.3 Primary and secondary outcomes

**Figure 2:**
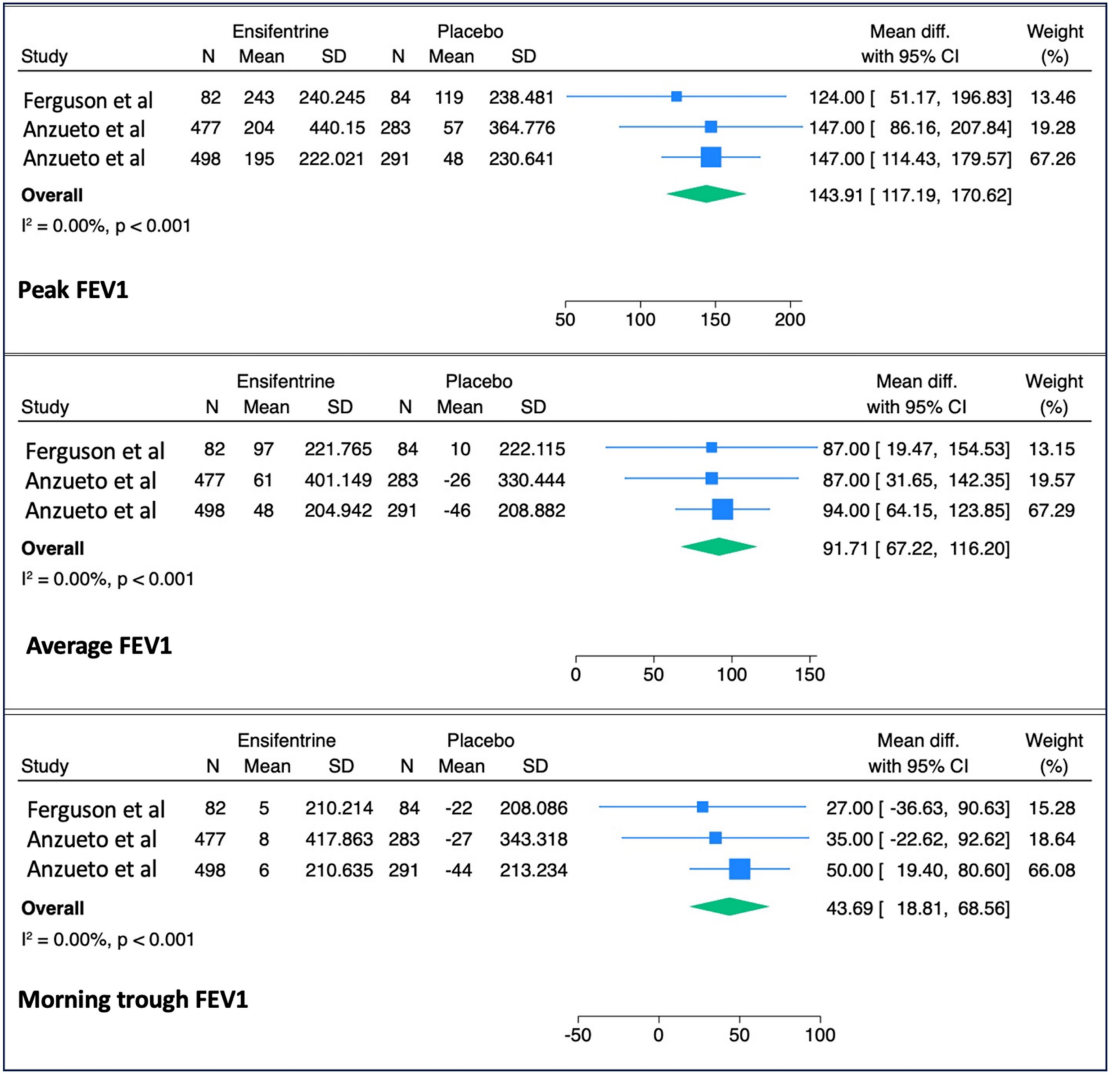
Expiratory volume outcomes. Pooled mean differences in forced expiratory volume in one second (FEV_1_) outcomes comparing ensifentrine to placebo, including peak FEV_1_, average FEV_1_, and morning trough FEV_1_. Peak FEV_1_ was defined as the maximum value within 3–4 h post-dosing, average FEV_1_ as the mean over 0–12 h, and morning trough FEV_1_ as the value measured prior to morning dosing.

**Figure 3:**
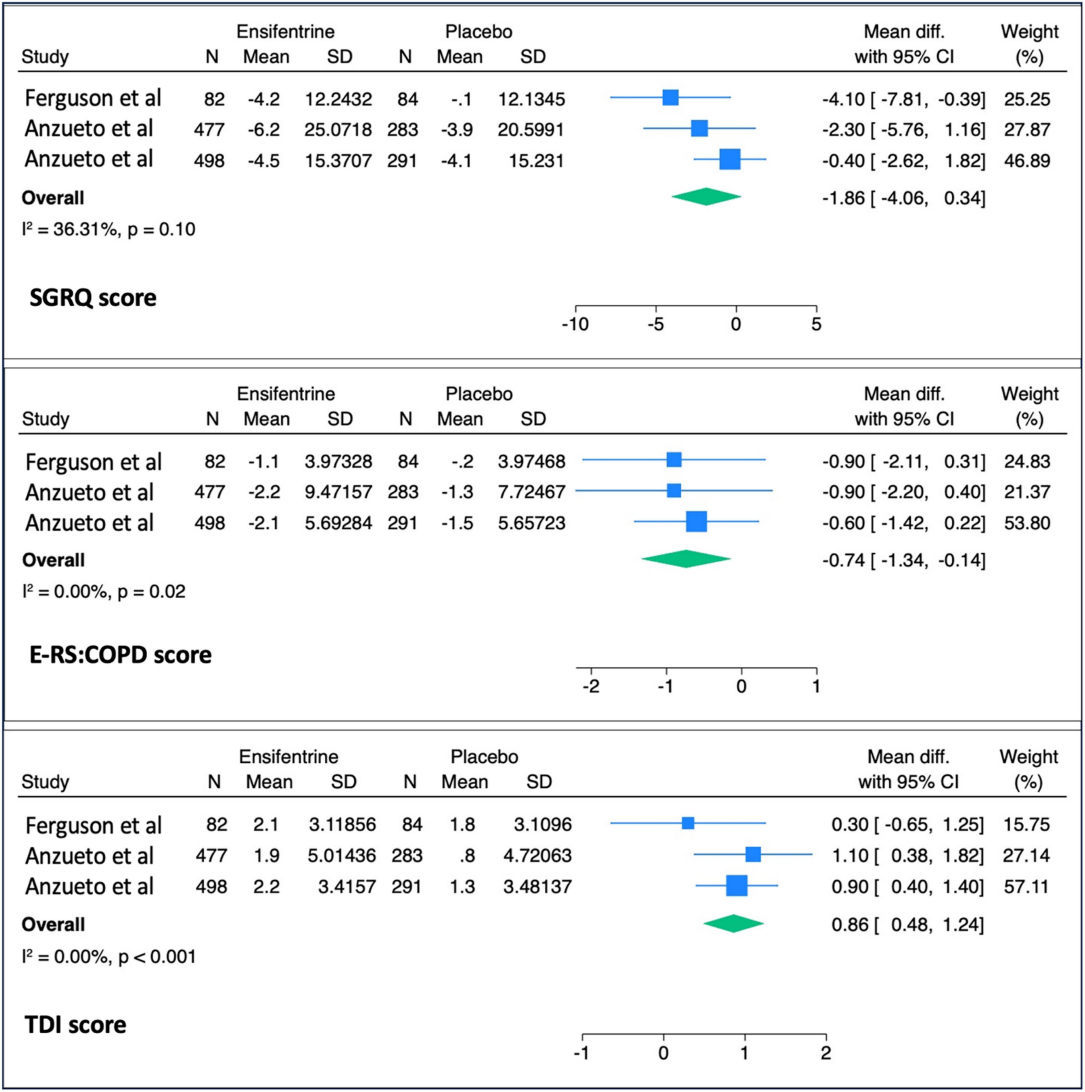
Secondary outcomes. Pooled analysis of secondary outcomes comparing ensifentrine versus placebo. These include changes in health-related quality of life and symptom scores, as assessed by the St. George’s Respiratory Questionnaire (SGRQ), the Evaluating Respiratory Symptoms in Chronic Obstructive Pulmonary Disease score (E-RS: COPD), and the Transition Dyspnea Index (TDI).

The mean difference in peak FEV_1_ (143.91 mL, 95% CI: 117.2–170.6), average FEV_1_ (91.7 mL, 95% CI: 67.2–116.2), and morning trough FEV_1_ (43.7 mL, 95% CI: 18.8–68.6) were all significantly increased when compared to patients treated with placebo (Figure 2). There was limited heterogeneity between included studies for these outcomes (*I*^2^ = 0.00, *p* < 0.001). For the secondary outcomes, ensifentrine significantly improved the E-RS: COPD (*p* = 0.02) and TDI scores (p < 0.001); however, SGRQ total score (*p* = 0.10) was not significantly improved (Figure 3).

### 3.4 Safety outcomes

**Figure 4:**
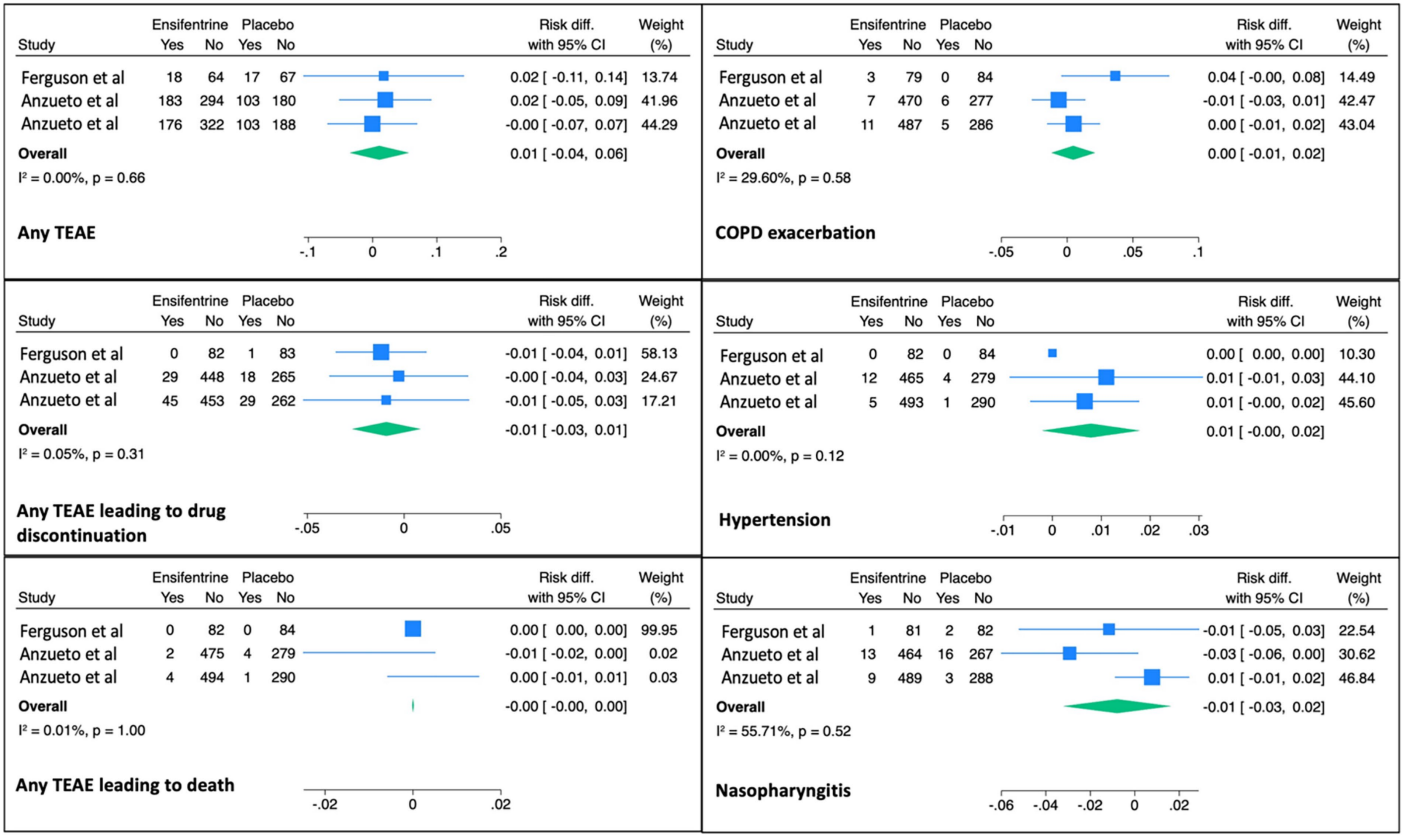
Safety outcomes. Pooled analysis of safety outcomes comparing ensifentrine to placebo, including the incidence of treatment-emergent adverse events (TEAEs), TEAEs leading to drug discontinuation or death, COPD exacerbations, hypertension, and nasopharyngitis. Outcomes are presented as risk differences with corresponding 95% confidence intervals.

Ensifentrine appeared to be a safe intervention with limited adverse effects. All categories of adverse effects were similar between the ensifentrine and placebo groups (Figure 4).

## 4 Discussion

We examined the pooled efficacy and safety of ensifentrine compared to placebo using clinical trials with comparable baseline characteristics and outcome measures. Ensifentrine demonstrated consistent improvements in both forced expiratory volumes and symptom scores, with limited heterogeneity.

The first clinical trial included in the systematic review was conducted by Ferguson et al. (23) to evaluate the effects of nebulized ensifentrine compared to placebo on various outcomes. The authors reported that ensifentrine significantly improved peak FEV_1_, average FEV_1_, and SGRQ total score than placebo on COPD patients who remained symptomatic while receiving muscarinic antagonists at the 4-week follow-up (23). Anzueto et al. (24) conducted ENHANCE-1 and ENHANCE-2, two multicenter, randomized controlled clinical trials designed to evaluate the efficacy of ensifentrine compared to placebo in treating moderate to severe symptomatic COPD. In ENHANCE trials, ensifentrine showed a significant improvement in average FEV_1_ than placebo over 12 weeks. In ENHANCE-1, ensifentrine also improved symptoms and quality of life as measured by E-RS and *SGRQ*, respectively, over a 24-week period.

Multiple doses of ensifentrine were tested for COPD in a dose escalation study conducted by Ferguson et al. (23). The doses tested ranged from 0.375 mg to 3 mg twice daily. Among these, the 3 mg twice daily dose was the most effective for improving airflow over a 12-h period (23). This dose also demonstrated greater efficacy in improving peak FEV_1_ at 4 weeks (14). These dose-finding studies informed the design of the ENHANCE trials, which utilized the 3 mg twice daily regimen (24). To limit heterogeneity, we included only the arms receiving ensifentrine 3 mg twice daily and placebo in this systematic review.

We observed no significant differences in treatment-related adverse effects between the ensifentrine and placebo groups. This finding is consistent with results from previous studies of ensifentrine. ENHANCE-1 and ENHANCE-2 assessed several adverse effects, including any treatment-emergent adverse event (TEAE), TEAEs leading to drug discontinuation or death, COPD exacerbation, hypertension, nasopharyngitis, and others. In the ENHANCE trials, the rates of adverse events were similar between the ensifentrine and placebo groups (24). Similarly, in a study conducted by Ferguson et al. (23), adverse events did not differ significantly between the ensifentrine and placebo groups. Overall, ensifentrine appears to have a favorable safety profile, with potential benefits in airflow and symptom scores.

PDE inhibitors are drugs that work by inhibiting *phosphodiesterase* enzymes and preventing the breakdown of the second messenger cAMP or cGMP in target cells (25). Roflumilast is the first oral selective *phosphodiesterase-4 inhibitor* approved for the prevention of COPD exacerbations. It provides beneficial effects on pulmonary inflammation and mucous hypersecretion; however, it is not widely recommended due to intolerable side effects such as weight loss and gastrointestinal disturbances (26–29). To reduce these adverse effects, inhaled PDE inhibitors were recently developed (28). Ensifentrine is an inhaled dual PDE3 and PDE4 inhibitor that offers both bronchodilatory and anti-inflammatory benefits. Compared to roflumilast, ensifentrine appears to have a more favorable safety profile, with fewer systemic side effects, supporting its potential as a better-tolerated therapeutic option in COPD management (10, 30).

Compared with previously published systematic review and meta-analyses on ensifentrine, our study offers several distinct methodological and clinical advantages. First, we categorized outcomes into clearly defined groups—primary (lung function), secondary (symptom and quality of life), and safety—which improves clarity and facilitates clinical interpretation. Second, we emphasized patient-centered endpoints, including TDI, St. SGRQ, and E-RS: COPD score outcomes that were either omitted or not fully explored in earlier meta-analyses. Third, prior studies by Fatima et al. (18) and Yappalparvi et al. (19) pooled data across multiple ensifentrine doses (0.75 mg, 1.5 mg, and 3 mg), including investigational and subtherapeutic regimens. While informative, such heterogeneity may reduce the direct applicability of their findings to clinical practice. More recently, Carvalhal et al. (20) identified a bell-shaped dose–response pattern, where therapeutic efficacy appears to peak at intermediate doses, with reduced benefit at lower or higher doses—possibly due to receptor desensitization (31). In this context, our exclusive focus on the approved 3 mg twice-daily dose offers clearer insights and greater clinical relevance. By isolating a single standardized regimen, our meta-analysis avoids confounding from dose variability and provides a more actionable synthesis aligned with current prescribing guidelines.

Multiple experimental studies have been conducted to investigate the potential mechanisms of ensifentrine in treating COPD and other lung diseases. Ensifentrine has been shown to elevate cAMP levels in human neutrophils and in isogenic human cystic fibrosis bronchial epithelial cells expressing wt-CFTR (CFBE41o-WT), indicating its ability to inhibit PDE3 and PDE4 to promote airway relaxation (32, 33). Ensifentrine alone caused relaxation of guinea pig airways and demonstrated synergistic bronchodilator effects when co-administered with salbutamol *in vivo* (34). Likewise, ensifentrine alone relaxed human bronchi and produced additive inhibition of airway smooth muscle contraction when combined with a beta 2 agonist and a muscarinic receptor antagonist, confirming its bronchodilator effect (35). On the other side, ensifentrine has been reported to exert anti-inflammatory effects in multiple experimental models. Ensifentrine led to a robust reduction in the production of pro-inflammatory cytokines in cystic fibrosis bronchial epithelial cells treated with interleukin-1β (IL-1β) (33, 36). Elevated levels of cytokines such as IL-1β and tumor necrosis factor alpha (TNF-*α*) have been shown to impair mucociliary function by disrupting ciliary activity, altering epithelial ion transport, and promoting excessive mucus secretion (37, 38). Therefore, this anti-inflammatory effect suggests that ensifentrine may improve mucociliary clearance in COPD patients. Moreover, ensifentrine significantly attenuated eosinophil recruitment in a guinea pig model following ovalbumin challenge (32). Similarly, treatment with aerosolized ensifentrine in an ovalbumin-sensitized guinea pig model significantly reduced the recruitment of total cells in BAL fluid, including neutrophils, monocytes, and eosinophils (39). Overall, ensifentrine exhibited protective effects by increasing cellular cAMP levels, inhibiting airway smooth muscle contraction, reducing pro-inflammatory cytokine levels, and decreasing the recruitment of immune cells.

This research may have substantial implications for COPD research and clinical practice. This systematic review of RCTs evaluated the efficacy and safety of ensifentrine compared to placebo for the treatment of COPD. This review found significant improvements in pulmonary function tests, quality of life, and symptom scores among patients treated with ensifentrine. In addition, this study focused specifically on the 3 mg twice-daily dose, which reflects the approved regimen and enhances clinical relevance.

This study also has several important limitations. The primary and secondary outcomes were limited to pulmonary function tests, quality of life, and symptom scores. Therefore, the effect of ensifentrine therapy on long-term patient-specific outcomes including rates of COPD exacerbations, hospital admissions, hospital length of stay, ICU length of stay, ventilator support, and mortality warrants further investigations. In addition, only three studies were eligible for inclusion in this systematic review due to strict criteria, with the majority of available evidence being preclinical. Although the included RCTs were generally well-designed, some variation in study quality and potential selection bias cannot be entirely ruled out. Lastly, the follow-up periods in the included studies were variable, ranging from 4 to 24 weeks.

## 5 Conclusion

We observed consistent and significant improvements in pulmonary function tests among patients treated with ensifentrine. These improvements were further supported by positive changes in two out of three quality-of-life and symptom scores, along with a favorable safety profile compared to placebo. Ensifentrine is a promising new therapy for the maintenance treatment of COPD. Future research is needed to fully evaluate the long-term, patient-specific effects of ensifentrine therapy, including its impact on COPD exacerbation rates and hospital admissions.

## Data Availability

The raw data supporting the conclusions of this article will be made available by the authors, without undue reservation.
